# Topics of the Month

**Published:** 1892-06

**Authors:** 


### TOPICS OF THE MONTH.

THE *American Lancet* informs the profession that arrangements for the approaching meeting of the American Medical Association are progressing satisfactorily. It asserts that everything that the Detroit profession can command will be placed at the service of the Association, and the united profession of Michigan cordially invites the attendance at the meeting of every member of the profession throughout the country. The *Lancet*, for itself, urges that every person interested shall come and remain during the entire meeting. We cordially thank the *Lancet* for its generosity, in not limiting its invitation merely to members of the Association, but in extending a cordial welcome to all physicians of standing throughout the length and breadth of the land. From our knowledge of the hospitality of Detroit and its citizens, we believe that much satisfaction and delight will follow an acceptance of its generous hospitality on this occasion.

---

THE *National Medical Review* has lately come to our table, and is a handsome periodical, to be issued monthly at the subscription price of one dollar a year. Dr. Charles H. Stowell, of Washington, D. C., is the editor, and it may be expected, from his knowledge and experience as an editor and publisher, that he will make the *Review* an established success. For a long time Washington has been without a medical periodical, which seems very strange in a city where learning, letters, and libraries are concentrated in a remarkable degree. Dr. Stowell prepares all the articles and paragraphs of his journal himself, selecting from all sources such topics as seem to merit publication, and then presents their substance in abstract. We bespeak a large measure of success for Dr. Stowell in his enterprise.

---

The address of Dr. George M. Gould at the graduating exercises of the Buffalo University Medical College was a manly plea for loyalty to legitimate medicine, and a terrible indictment against all forms, modes, and devices of quackery. We wish we had space to publish it in full, but can only give a few selected pearls from this choice string :

. . . . . . . . . . . . . . . . . . . .

Quackery may be likened to a poor artificial eye—everybody can see through it except the patient.

. . . . . . . . . . . . . . . . . . . .

To be explicit and detailed, let me counsel a few "don'ts."

1. Don't be in a hurry for success.

2. Don't consult or fraternize with quacks of any kind or degree.

3. Don't be afraid of speaking out your denunciation of quackery, regardless of the loss of a few possible patients and the charge of jealousy.

4. Don't support medical journals run in the interests of the advertisers, journals that are muzzled, that are conciliatory, or non-denunciatory of quackery.

5. Don't sign a single certificate so long as you live as regards special, proprietary, or secret preparations.

6. Don't write a medical article in which such preparations are praised, or even mentioned.

7. Don't accept commissions or presents from druggists, manufacturers, opticians, or surgical instrument dealers.

8. Don't let any professional allusion to yourself, your opinions, or your work, get into the lay newspapers. Don't be a sneak advertiser, a "newspaper doctor."

9. In your own righteous wrath against quacks outside of the profession, don't forget that there are many in the profession, and that they are the most despicable,—true wolves in sheep's clothing. I would rather be the "Wizard King of Pain," and buy affidavits of impossible cures at twenty dollars each, than a respectable hypocrite indirectly or secretly hobnobbing with newspaper reporters and supplying them with data.

. . . . . . . . . . . . . . . . . . . .

Reflect, thirdly, that all the world over, every physician, whenever asked, gives his services to the poor without demand or without hope of compensation. Would not a lawyer, or a locksmith, think one crazy if it were proposed that he should give a large share of his time and service for nothing?

Carry the thought on. The entire tremendous labor, for the benefit of the community, of keeping up the enormous hospital work of all the world's cities, is borne by physicians without a cent of pay. Are there, for example, thousands of similar institutions where the poor, free of charge, can get legal counsel and help? Is there one such?

. . . . . . . . . . . . . . . . . . . .

---

The Buffalo General Hospital has lately issued its twenty-third annual report, being for the year 1891. It is an interesting pamphlet, giving a résumé of the work of the hospital for the year, and contains a report of the training-school for nurses.

---

The Arnot-Ogden Memorial Hospital, of Elmira, presents a neat and interesting report for the year 1891. The buildings, which are handsome modern structures, together with the hospital grounds, stand as a memorial of the Arnot and Ogden families, and this, in our opinion, is one of the most substantial, lasting, and philanthropic ways in which family names can be perpetuated. We wish that more of this same kind of philanthropic spirit might pervade the wealthy people of our country.

---

The annual report of the Board of Managers of the Buffalo Historical Society, for the year 1891, is a handsome brochure of sixty-six pages, that does credit to the officers and members of this established institution. A handsome photogravure of the monument and Brown's statue of Red Jacket, erected in Forest Lawn Cemetery by the Buffalo Historical Society, faces page sixteen of this report, and is an index of what can be done in the way of organized efforts to perpetuate historic memories. We hope this interesting report will be extensively read by our citizens, and that a large increase of membership will come to the society during the present year.

---

Mrs. Jane C. Stormont, widow of the late Dr. David W. Stormont, of Topeka, Kansas, has erected the Stormont fund for the establishment and maintenance of a medical library in the City of Topeka. Mrs. Stormont has given $10,000 to this enterprise, one-half to be a permanent fund, and the remainder to be used in the purchase of books. The profession of medicine everywhere commends the spirit which has actuated this generous-hearted and noble type of American womanhood, and looks forward to the wholesome influence that her example will exert.

---

The question of literary theft has always been an nteresting as well as a disturbing one. The St. Louis *Medical and Surgical Journal*, in a late number, says that by a recent decision of a French Tribunal a medical journal is not permitted to publish an extract from another without giving due credit. We think that this decision might properly be applied to the administration of American law, and would have a beneficial influence on many medical journals in this country. The *Medical and Surgical Journal* cites a case where a guilty party was condemned to pay a large sum for every article cribbed, and to publish the findings of the court. If some such law were enacted here, its influence would be salutary.

---

A Famous Malpractice Case.—Most of our readers in this vicinity have been made aware, by the daily newspapers, of the fact that an action has lately been tried in the courts of Buffalo, entitled Kemball vs. Cary. The details of this action, which has been tried twice, the last time by a struck jury, and resulted in a verdict of no cause of action, are familiar to our readers. It is not, therefore, our purpose nor our duty to take up space in these columns with even a résumé of the case, but as observers of medical affairs in this city and vicinity we are interested in the prosperity and honor of the medical profession, and we wish to record our approval of the finding of the jury in the last trial of this now somewhat famous case. There cannot be two opinions among careful observers as to the justice of the verdict, and we congratulate Dr. Cary, the defendant, upon the sterling and successful fight that he has made for himself, as well as for his professional brethren, in contesting this case. The lesson to be drawn is, that when a physician engages in a case with good intent, and gives his time and skilful attention to its management, he must not be mulcted in damages by reason of some technicality of law or through the machinations of designing men.

---

WE HAVE received a list of an extensive line of standard and pharmaceutical preparations, the modern medicaments manufactured by Parke, Davis & Co., of Detroit, which they have recently issued to send out to the profession. One of the notable features of this exceedingly attractive and well-arranged list is the representation by forty engravings of the laboratories at Detroit, and the branches at New York, Kansas City, and Wakeville, Ont., of this famous house. These engravings comprise views of the interior and exterior of the laboratories, offices, and sections of the department at Detroit, and altogether it appears the most complete list ever published of the products of this well-known house. It will be welcomed by every friend and patron everywhere, and will be sent to all physicians on application.

---

THE sudden death of Sir Morell Mackenzie, which occurred at his home in London, February 3, 1892, has afforded occasion for many tender expressions of affectionate regard for this great leader from his American friends and admirers. Among them we select the following beautiful monody, from the pen of Dr. William Porter, of St. Louis, as a most touching tribute to the life and character of our deceased friend :

SIR MORELL MACKENZIE.

The master rests. After the day of toil
An urgent message came to him, and he
Well used to sudden calls, in quiet haste,
With kind good-night went out and all was still.
And now his work is done ; to him no more
Will come the suffering ones and those who need
The helping hand and words of goodly cheer.
His last response completed all his work.
O strong and gentle heart, ours is the loss
Who knew thee well—and knowing loved thee more.
Ours is the loss and thine the great reward.
We crown thee victor, O thou kingly dead.

We have heretofore (Volume XXIX., page 592, April, 1890,) called attention to the frequent mispronunciation of "gynecology." We return to the subject once more, because we have been unable to discover any considerable improvement in the pronunciation of this much abused word. In a paper upon Medical Orthoepy, by Dr. J. F. Oaks, of Chicago, we find the following:

"*Of the Consonants.*—The consonants in Latin, according to the English method, are usually pronounced like the corresponding English letters in the same situation; *e. g.*, C has the sound of s, and G the sound of j, before e, i, and y, and the diphthongs æ and œ. Since it is reasonably certain that C and G were always hard in the language of the ancient Romans and Greeks, some authors contend that we should assign to these letters in words of Greek or Latin origin the same hard sounds found in the original language. The premises are incorrect and inconsistent with general usage. We say ġeometry, ġenesis, and not ḡeometry, ḡenesis, and yet the latter is no more unreasonable than to say ḡȳ′necology. The orthography of the latter word in Latin is G.y.n.e.c.o.l.o.g.i.a., and is pronounced according to the Roman method, ḡy̆necolo′-ḡia, and in conformity with the English method gy̆n′ ′ecolo′ ġia. We have, however, in the word gynecology the English analogue, conforming to English orthography, and is pronounced ġy̆n-ecol′ oġy, like ġy̆narchy and ġy̆neocracy of similar analogy and etymology."

This would seem to settle the matter authoritatively on a question where there ought never to have been any difference of opinion, since there is but one right way to pronounce this word.

---

A comprehensive series of World's Congresses will be held at Chicago during the season of the Columbian Exposition of 1893, among which will occur the World's Congress of Pharmacists. This Pharmaceutical Congress will be under the supervision of a special committee, of which Oscar Oldberg is chairman, and a comprehensive sketch of the plans of the congress has been sent out to the public press and all interested in the success of pharmaceutical affairs. We need not say that this subject is one of vital interest to the profession of medicine, as well as to the patrons of pharmacy, and we are pleased to record our approbation of the comprehensive scheme which is outlined in the address of the committee, as well as to give it the encouragement of our kindly words and sanction.

The bulletin of the Tennessee State Board of Health for January complimented the State of Michigan for its advanced standard and progressive excellence in regard to its public health affairs. The bulletin commends the action of the Michigan State Board of Health in holding frequent sanitary conventions in different parts of the State, and asks other States to use similar methods. There can be no doubt that the system which Michigan has inaugurated and carried on so successfully, is one that every State may do well to imitate, for it serves to educate the masses with reference to the importance of hygienic and sanitary measures.

---

The *Medical Missionary Record*, for April, states that Mrs. Howard, of Buffalo, has given $2,000 to meet the cost of erecting a wing to the Westminster Hospital, Oroomiah, Persia. We wish the *Record* had stated Mrs. Howard's full name, that we might give her proper credit for this philanthropic act, and which, to quote the language of the *Record*, must be a pleasant satisfaction to herself, and a great blessing to the poor suffering women of Persia. It seems as though the women were outstripping everybody in the race for philanthropic work.

---

The Students' Exemption Bill, which has created so much concern on the part of the friends of medical education reform, did not become a law after all. It passed the State Senate, and under the pressure of organized effort on the part of medical students, backed by very many members of the medical profession who ought to have been in better business, it came very near favorable action in the house. It is probable that the even vote in the Committee on Public Health, which practically killed the bill, was largely due to the influence of the Speaker of the Assembly, Dr. R. P. Bush, who attended the hearings, and who is well known for his friendliness to the State Examiners' law, as well as for his opposition to any measures that seek to render its effects nugatory. We are pleased to notice that the *Post-Graduate* gives Dr. Bush full credit for his manly action in this matter, as well as for his opposition to all medical laws that were offered in the late legislature, whose objects were to degrade legitimate medicine. In this connection it may not be improper for us to remark that the biographical sketch of Speaker Bush, that we published in the February number of this journal, has been extensively copied, either in whole or in part, by the medical journals throughout the country, some of which have been generous enough to credit the JOURNAL with what it said.

---

THE abatement of nuisances seems to be in order, and we note that considerable progress is making by the municipal authorities in regard to this subject. We beg, therefore, to suggest that the church bell nuisance be not overlooked. This relic of a past age, when the people worshipped on high hills and in low valleys, ought not to be tolerated in a populous city where health and even life may hang upon a thread in the vicinage of the awful din that the conglomerate sounds of numerous bells create, especially if they are hung in one tower. It is in vain that appeals have been made to certain religious sects on the subject, and in more than one instance, do we know, where one of these sects has refused to stop the ringing of bells even when death was nigh at hand in their immediate vicinity. We hope the high hand of municipal authority will stretch itself out, and lay it down heavily on this nuisance that menaces comfort and sometimes even life.

---

THE next examinations of candidates for the license to practice medicine in this State, will be held on June 14th, 15th, 16th, and 17th, in the following-named cities: New York, 410 E. Twenty-sixth St.; Albany, at the High School; Syracuse, at the High School; Buffalo, at the High School. The examinations will be conducted as follows: Tuesday, June 14th, on Anatomy, Physiology, and Hygiene; Wednesday, June 15th, on Chemistry and Surgery; Thursday, June 16th, on Obstetrics, Pathology, and Diagnosis; Friday, June 17th, on Therapeutics.

Applications should be made to the State Board of Regents at once for permission to appear at these examinations.

---

THE Section on Medical Pedagogics of the Pan-American Medical Congress of 1893 will be one of the most important subdivisions of that great Congress. It will have to do with the methods of teaching medicine in all its various phases and aspects, and will deal with the question in a practical, and not theoretical, manner. This section will be presided over by Dr. D. B. St. John Roosa, of New York, than whom we know no more competent man to administer the important affairs of this very important section.

The Fresh Air Mission of Buffalo has decided that if proper encouragement is given, it will this year add to its activities a hospital for the treatment of cholera infantum. In the Journal for September, 1889, Vol. XXIX., page 123, we called attention to a hospital for a similar purpose, which is located at Charlotte, and which was organized and is maintained by the benevolent citizens of Rochester. We suggested the propriety of establishing a similar hospital in Buffalo. The fruits of our suggestion appear to have taken root, and a public meeting has been held, which was addressed by Dr. E. M. Moore and others, of Rochester, who were the projectors of the Charlotte Hospital. It is to be hoped that there will be no lack of spirit and interest in this subject, and that the funds necessary to maintain such a hospital will be forthcoming. It is one of the most noble charities that philanthropic people can establish.

---

The Buffalo Academy of Medicine, the forming of which we brought to the notice of our readers in the April issue of the Journal, was made an accomplished fact at a meeting of the associate societies, held at the Y. M. C. A. Building, Tuesday evening, May 17, 1892. After adopting the constitution and by-laws that had been carefully prepared by the representative committee, nominations of officers and other arrangements were made in preparation for the annual meeting, Tuesday, June 21, 1892, at which time the election will be held. It will be conducted on the Australian ballot system, and the following nominations have been made: For president—Dr. Thomas Lothrop, Dr. DeLancey Rochester, Dr. C. C. Wyckoff, Dr. John Cronyn, and Dr. C. G. Stockton. For trustees—Drs. F. W. Bartlett, J. W. Putnam, A. E. Persons, A. Dagenais, Benjamin Long, Roswell Park. For secretary—Dr. W. C. Krauss. For treasurer—Drs. Eugene A. Smith, H. E. Hayd.

